# Gastrointestinal parasites of cats in Egypt: high prevalence high zoonotic risk

**DOI:** 10.1186/s12917-022-03520-0

**Published:** 2022-11-29

**Authors:** Ibrahim Abbas, Moustafa Al-Araby, Bassem Elmishmishy, El-Sayed El-Alfy

**Affiliations:** Parasitology Department, Faculty of Veterinary Medicine, Mansoura University, Mansoura, 35516 Egypt

**Keywords:** Cat, Egypt, Zoonosis, Toxocara, Hookworms, Opisthorchis, Cystoisospora, Toxoplasma gondii

## Abstract

**Background:**

Several gastrointestinal parasites that infect cats pose potential health threats for humans and animals. The present study is the first to report gastrointestinal (GIT) parasites in feces of stray cats from Gharbia governorate, Egypt. Findings were combined with those published in the earlier surveys from various Egyptian governorates, and various meta-analyses were conducted to underline the parasitic zoonoses from cats in Egypt.

**Results:**

Out of 143 samples tested in Gharbia, 75 (52.4%) were found infected with 13 different parasites. Co-infections were observed in 49.3% of positives. Several parasites were detected, e.g., *Toxocara cati* (30.0%), *Toxascaris leonina* (22.4%), hookworms (8.4%), taeniids (4.2%), *Strongyloides* spp. (2.1%), *Physaloptera* spp. (2.1%), *Alaria* spp. (1.4%) and *Dipylidium caninum* (0.7%). *Opisthorchis*-like eggs were found in a single sample being the first report from cats in Africa. Oocysts of 4 coccidian parasites were identified, and a few *Toxoplasma gondii*-like oocysts were detected in 2 samples (1.4%). Results of the meta-analysis illustrated that occurrence of *T. gondii* oocysts in feces of cats from Egypt may have been overestimated in earlier studies; 1432 cats have been tested and displayed a 5 times higher pooled prevalence (11.9%) than the published global pooled prevalence for *T. gondii* oocysts in cats. This overestimation might have occurred because some small-sized oocysts that belong to other coccidian parasites were mis-identified as *T. gondii*. *Toxocara cati* had a high pooled prevalence (22.5%) in cats from Egypt, which is even greater than the published pooled prevalence in cats globally; however, several reports from Egypt have neglected the role of *T. cati* in human toxocarosis. *Dipylidium caninum* displayed also a high prevalence (26.7%).

**Conclusion:**

Several zoonotic parasite species have been found in stray cats from Egypt, raising concerns about the risks to the Egyptian human population as well as environmental contamination. Prompt surveillance supervised by the government and accompanied by data dissemination will be helpful for developing effective control strategies.

**Supplementary Information:**

The online version contains supplementary material available at 10.1186/s12917-022-03520-0.

## Background

The estimated global cat population is 700 million comprising 480 million living as strays and 220 million as pets (https://www.carocat.eu). Cats can be infected with various parasites including those inhabiting the gastrointestinal tract (GIT), which represent a growing concern from both veterinary and public health perspectives because of their impact on cat health as well as their potential to infect humans [1, 2]. In several regions worldwide, cats live mostly as strays receiving no or little veterinary care, and stray cats often have a high prevalence of GIT parasites suggesting high health risks for humans living in these regions [3].

While many studies have been conducted to investigate the GIT parasites infecting cats in Europe [2, 4–6], a little is known about feline GIT parasites and their zoonotic impacts in Africa [7]. However, a recent study hypothesized that cats were likely domesticated in an African country (Egypt) 4000 years ago [8, 9]. A few studies have been conducted in Egypt mostly on a small population of cats from limited regions in the country, e.g., Cairo and Giza; however, reports from some Egyptian governorates e.g., Gharbia governorate, are lack.

Cats share dogs some GIT parasites that can cause serious disease in humans, e.g., hookworms, *Giardia* and *Cryptosporidium*. Evaluating the whole situation of these parasites in dogs and cats is crucial to demonstrate how dogs or cats contribute to the epidemiology of these zoonotic parasites in a region. For example, zoonosis caused by members of the genus *Toxocara* (*T. cati* from cats and *Toxocara canis* from dogs) is common worldwide. The relative contribution of both species in human toxocarosis is largely unclear due to lack of valid serological assays for differentiation as well as seldom recovery of the causative larvae [10]. Recent studies analysing the global situation of *Toxocara* species have shown that the prevalence in cats is significantly higher than that in dogs, suggesting a substantial role of cats in human toxocarosis [11, 12]. In Egypt, toxocarosis is quite common among humans [13]; many clinical cases have been documented in the recent years and the majority of these cases have been attributed to *T. canis* as the causative agent [14]. This highlights the need for establishing a comprehensive study, based on the published data from various governorates, analysing the situation of *Toxocara* spp. in cats and dogs from Egypt, which will aid in understanding the epidemiology of human toxocarosis in this country.

The objective of the present study was to determine, for the first time, the prevalence of various GIT parasites infecting stray cats in Gharbia governorate, Egypt. In addition, efforts were made to highlight the potential relevance of GIT parasites infecting cats as a source of parasitic zoonoses in Egypt within the frame of the One Health concept.

## Materials and methods

### Samples collection and laboratory processing

**Fig. 1:**
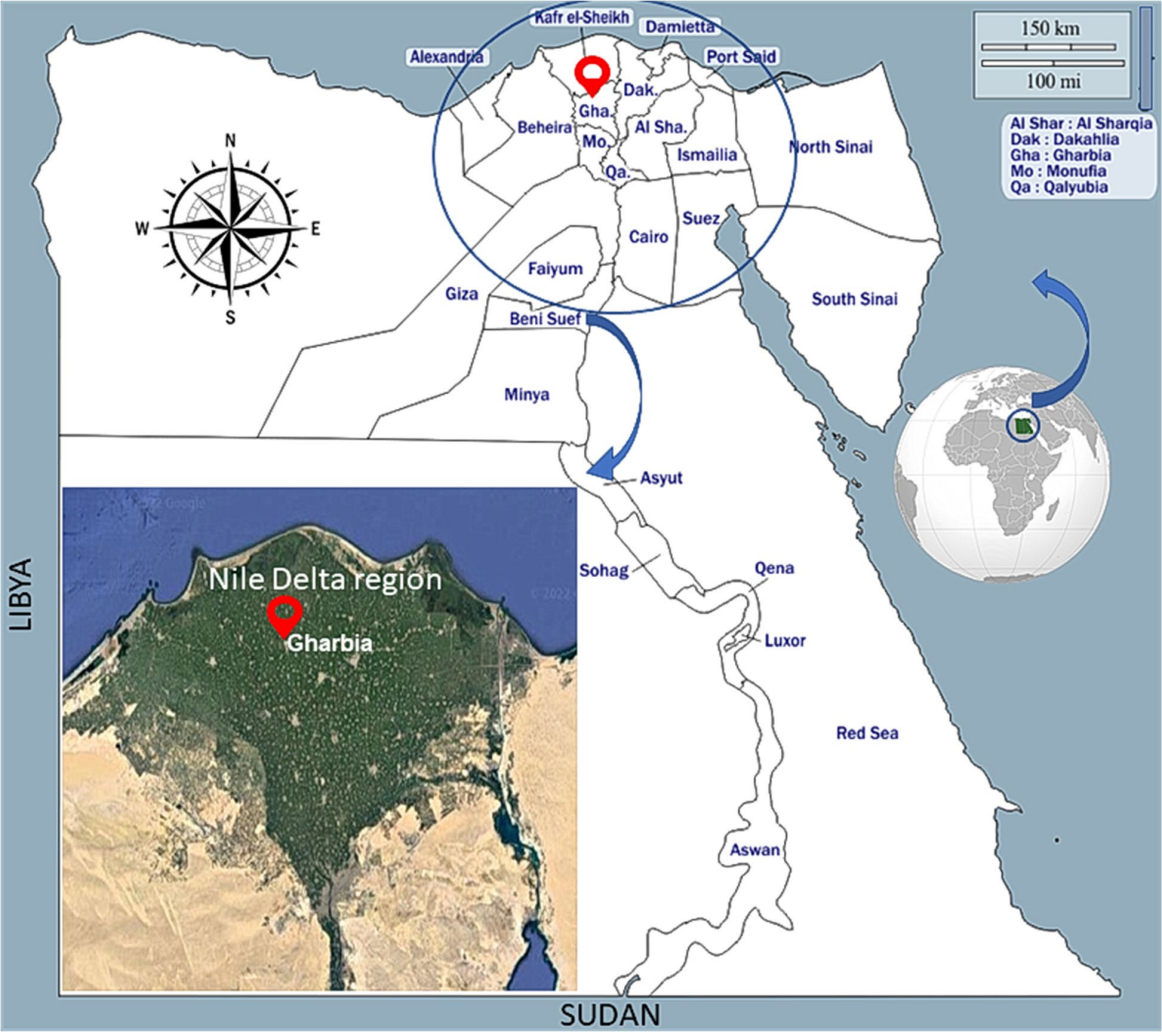
Map of Egypt illustrating the study area. Source of original map before modifications: https://d-maps.com/carte.php?num_car=25356&lang=en

A total number of 143 fecal samples of stray cats were collected over a year (January—December 2021) from different rural areas in Gharbia governorate located in the Nile Delta, Egypt (Fig. 1). The total area of Gharbia is approximately 2000 km^2^. Around 5 million people are residing this governorate, with a very high population density ratio. The Nile Delta has a semi-desert climate with hot dry summers (temperature 31—34 °C in average) as well as warm (9 -19 °C) slightly rainfall (100–200 mm) winters [15, 16].

Samples were collected within 15 batches (each composed of 5–15 samples) from different areas. All cat fecal samples available to us from the sampled areas were collected. Many samples were collected in a fresh state after watching the cats depositing and burying their feces in soil around resident houses or animal farms. Other samples were collected from different sandy spots and displayed different degrees of dehydration due to their earlier deposition. Samples were stored at 4 °C immediately after collection and treated under strict hygienic conditions. In the next day, samples were grossly inspected and examined under the stereoscopic microscope (M6C-9, USSR) to identify any tapeworm segments or adult nematodes present, then were tested by means of routine sedimentation followed by the modified Wisconsin sucrose (specific gravity > 1.27) flotation technique [17, 18]. The recovered parasitic stages were identified morphologically based on pervious keys [19–21]. Coccidian oocysts recovered during examination were invitro-sporulated at room temperature using 2.5% potassium dichromate. No further procedures for delimitation of some species (e.g., hookworms) were conducted. Micrographs were captured using a binocular microscope (Carl Zeiss, Oberkochen, Germany) equipped with 12 MP camera (AmScope®, USA). Results were statistically analysed using a chi-square test. The 95% confidence intervals of a proportion including continuity correction and odds ratios were calculated using www.vassarstats.net. Differences with *p* < 0.05 were considered significant.

### Data collection and analysis

A systematic electronic search was conducted to collect all published articles on GIT parasites infecting cats in Egypt. The international databases (Google Scholar, PubMed, Scopus, and Science Direct) were searched by two of the authors (EE and BE) using the following keywords: cat, feline, Egypt, stray, gastrointestinal parasites, faeces*, Toxocara cati*, hookworms, taeniids, *Dipylidium caninum*, *Strongyloides*, *Toxoplasma gondii*, *Sarcocystis*, *Isospora*, *Giardia* and *Cryptosporidium*. The Boolean operators “AND” and “OR” were used to connect the entry terms. Websites of the local databases: Egyptian knowledge bank (http://www.ekb.eg) and the Egyptian university libraries consortium (http://srv4.eulc.sdu.eg/eulc_v5/libraries/start.aspx) were consulted to collect articles published in local journals. Libraries of Faculty of Veterinary Medicine, Mansoura University, as well as Parasitology Department, Faculty of Medicine, Ain Shams University, Egypt were screened to collect articles published in local journals that had no electronic copies. The collected articles were screened for eligibility by EE and BE and any discrepancies were discussed with the first author (IA). Articles were defined eligible when the study 1) was conducted on cats from Egypt, 2) available in full text, 3) published as a research article, 4) found positive samples for any GIT parasite in cats, and 5) had a defined number of tested and positive samples. Articles that did not meet these criteria were excluded. For example, articles on non-GIT parasites in cats and articles available only as conference or proceeding abstracts as well as review articles.

Two of the authors (EE and BE) independently extracted important findings of the eligible studies, e.g., study sub-region, sample size, number of positives, diagnostic methods, parasites detected and mode of life (stray/household). Data were tabulated in Microsoft Excel spreadsheets, then used for various meta-analyses conducted in the present study using the software OpenMeta[Analyst] [22]. All analyses were computed based on a 95% confidence interval, and the pooled estimates that represented prevalences of the included parasites were determined employing the random effects model coupled to the DerSimonian-Laird method. The heterogeneity among the included studies was calculated using the *I*^*2*^ statistic and the heterogeneity were considered high when *I*^*2*^ values exceeded 50%. Subgroup analyses were conducted to investigate variations in prevalence of the included parasites according to the Egyptian regions, mode of life of the tested cats (stray or household) and to the detection method used (fecal examination or intestinal necropsy). Publication bias was not assessed in the present study because it is not considered relevant for prevalence studies [23].

## Results

### Prevalence of GIT parasites infecting stray cats in Gharbia

Seventy-five (52.4%) out of 143 faecal samples collected from stray cats in Gharbia, had eggs/oocysts of at least one parasitic species. Single (26.7%; *n* = 38) and mixed (25.9%; *n* = 37) infections were detected almost in equal rates. Samples from 28 (19.6%) and 8 (5.6%) cats had dual and triple infections respectively, whereas a single sample (0.7%) had quadruple infection (Table 1).

**Table 1:** Infection patterns among individual fecal samples that tested positive for GIT parasites in 143 stray cats in Gharbia governorate

Infection pattern	No. infected (%)	OR (95% CI)	*P*-value
Single infection	38 (26.6)	Ref	Ref
Dual infection	28 (19.6)	1.48 (0.85—2.58)	0.16
Triple infection	8 (5.6)	6.10 (2.73—13.64)	< 0.0001
Quadruple infection	1 (0.7)	51.39 (6.94—380.32)	1 (0.64)
Total	75 (52.4)	NE	NE

*OR* Odds ratio, *CI* Confidence Intervals, *Ref* Reference group compared with other groups, *NE* Not estimated

OR and *P*-value were calculated according to method described by (http://vassarstats.net/)

**Fig. 2:**
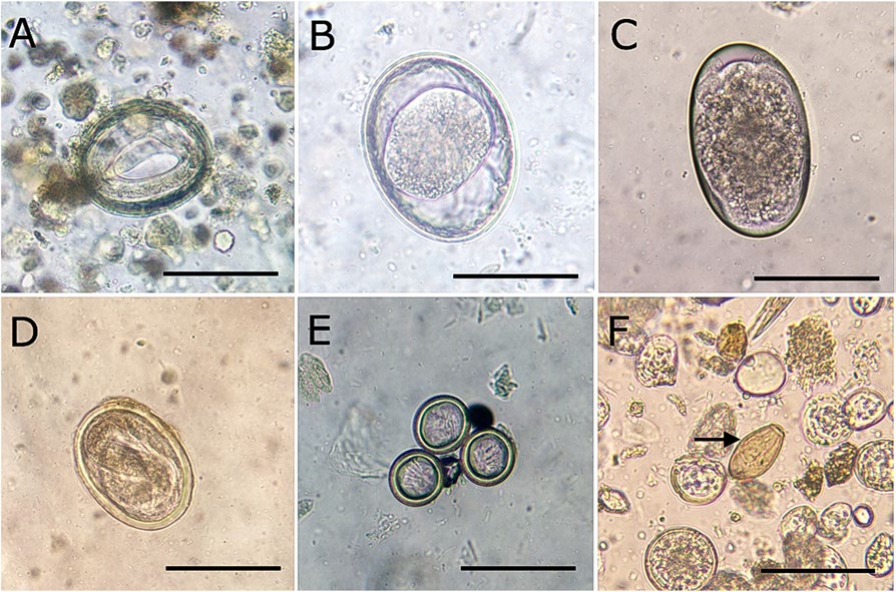
Micrographs of helminths eggs isolated from stray cats in Gharbia governorate, Egypt. **A** *T. cati* larvated egg, **B** *T. leonina* embryonated egg, **C** Hookworms egg, **D** *Physaloptera * spp. egg, **E** Taeniid eggs, **F** *Opisthorchis-*like egg

Eggs/oocysts of 13 GIT parasites were detected in the tested samples comprising 9 helminth species (5 nematodes, 2 tape worms and 2 flukes) as well as 4 protozoan parasite species (Table 2). *Toxocara cati* was the most frequently detected parasite (*n* = 43; 30.0%) followed by *Toxascaris leonina* (*n* = 32; 22.4%). *Toxocara cati-T. leonina* combination represented the most frequently detected form of dual infections (see Additional file 1). Eggs of other helminths were detected at lower rates: hookworms (8.4%), taeniids (4.2%), *Strongyloides* spp. (2.1%), *Physaloptera* spp. (2.1%), *Dipylidium caninum* (0.7%) and *Alaria* spp. (1.4%) (Fig. 2). *Opisthorchis*-like eggs were interestingly detected in a single sample. Eggs measured 20–30 X 10–20 µm, yellow brownish-coloured and contained mature miracidia. Eggs were operculated and had prominent opercular shoulders and abopercular knob (Fig. 2F). Other helminth eggs (e.g., *Hymenolepis diminuta*, Anoplocephalid spp. and poultry ascarids) were occasionally observed, but were considered as spurious parasites.

**Table 2:** Prevalence of various GIT parasites detected in 143 fecal samples from stray cats in Gharbia governorate, Egypt

Parasite	No. positive (%)	OR (95% CI)	*P*-value
*T. cati*	43 (30.0)	Ref	Ref
*T. leonina*	32 (22.4)	1.49 (0.87—2.53)	0.14
Hookworms	12 (8.4)	4.68 (2.34—9.34)	< 0.0001
*Strongyloides* spp.	3 (2.1)	20.0 (6.03—66.3)	< 0.0001
*Physaloptera* spp.	3 (2.1)	20.0 (6.03—66.3)	< 0.0001
Taeniids	6 (4.2)	9.79 (4.01—23.89)	< 0.0001
*Dipylidium caninum*	1 (0.7)	60.88 (8.24—449.38)	< 0.0001
*Alaria* spp.	2 (1.4)	30.22 (7.15—127.65)	< 0.0001
*Opisthorchis-*like eggs	1 (0.7)	60.88 (8.24—449.38)	< 0.0001
*Sarcocystis* spp.	10 (7.0)	5.70 (2.73—11.89)	< 0.0001
*C. rivolta*	7 (4.9)	8.33 (3.6—19.28)	< 0.0001
*C. felis*	6 (4.2)	9.79 (4.01—23.89)	< 0.0001
*T. gondii-like* oocysts	2 (1.4)	30.22 (7.15—127.65)	< 0.0001
Total	75 (52. 4) ^*^	NE	NE

*OR* Odds ratio, *CI* Confidence Intervals, *Ref* Reference group compared with other groups, *NE* Not estimated

OR and *P*-value were calculated according to method described by (http://vassarstats.net/)

^*^Total number of infected samples including samples with mixed infections

**Fig. 3:**
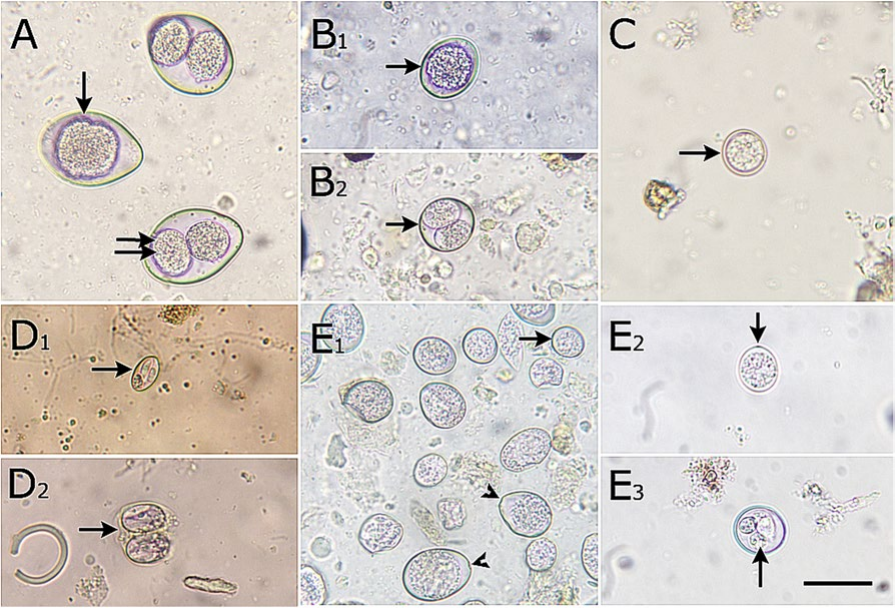
Micrographs of protozoan oocysts isolated from stray cats in Gharbia governorate, Egypt. **A** *Cystoisospora felis* unsporulated (single arrow) and sporulated (double arrows) oocysts, **B** *Cystoisospora rivolta* unsporulated (**B1**) and sporulated (**B2**) oocysts, **C** *T. gondii-*like oocyst, **D** *Sarcocystis* spp. sporocyst (**D1**) and oocyst (**D2**), (**E1-2**) unsporulated different *Eimeria* spp. oocysts, (**E3**) sporulated *Eimeria* spp. oocyst

The coccidium *Cystoisospora* (9.1%) was the most frequently detected protozoa. Two *Cystoisospora* spp. were identified; *Cystoisospora felis* (4.2%) and *Cystoisospora rivolta* (4.9%). One sample displayed mixed infection with the two species. Infections were mostly less intense; however, a *C. felis*-infected sample had high oocyst count (18,400 oocyst per gram). Oocysts of both species were found unsporulated, but occasionally early sporulated oocysts containing 2 sporoblasts were observed in old deposited fecal samples. Oocysts of *C. felis* (*n* = 40) were ovoid, large (30–50 X 25–35 μm) with a length–width ratio of 1.3–1.4 (Fig. 3A). Oocyst walls were 1.3 μm thick and smooth. No micropyle, polar granule or oocyst residual body were detected. Sporocysts measured 20–25 X 15–20 μm. *Cystoisospora rivolta* oocysts (*n* = 20) were ovoid to somewhat ellipsoidal, medium-sized (18–25 X 15–25 μm) with a length–width ratio of 1.1–1.3, and had thin (0.5 μm) smooth walls. No micropyle, polar granule or oocyst residual body were observed. Sporocysts were broadly ellipsoidal and measured 13–15 X 10–12 μm (Fig. 3B).

A few *T. gondii*-like oocysts (3–5) were detected in 2 samples. Oocysts were small-sized (11–13 μm), spherical to subspherical, filled with the sporont and had double-layered colourless oocyst walls (Fig. 3C). No polar granules were observed. Unfortunately, no *T. gondii*-like sporulated oocysts were recovered from these 2 samples after invitro sporulation, most likely due to the limited oocyst number. *Sarcocystis* spp. (7.0%) was also noticed, frequently as individual oval sporocysts (9–11 µm) with 4 sporozoites (Fig. 3D1), but occasionally as fragile thin-walled sporulated oocysts of different sizes (14.5- 17 µm) (Fig. 3D2). Unsporulated oocysts of different sizes (12–35 μm) were also observed in 10 additional samples. Oocysts were spherical, subspherical, oval or ovoid, and had micropyles, which in some oocysts was shallow and barely seen. After sporulation, tetrasporocystic dizoic oocysts were recovered, confirming their identity as *Eimeria* spp. This type of oocysts was considered as spurious parasites (Figs. 3E1-3).

### Overall prevalence of the most common GIT parasites infecting cats in Egypt

**Fig. 4:**
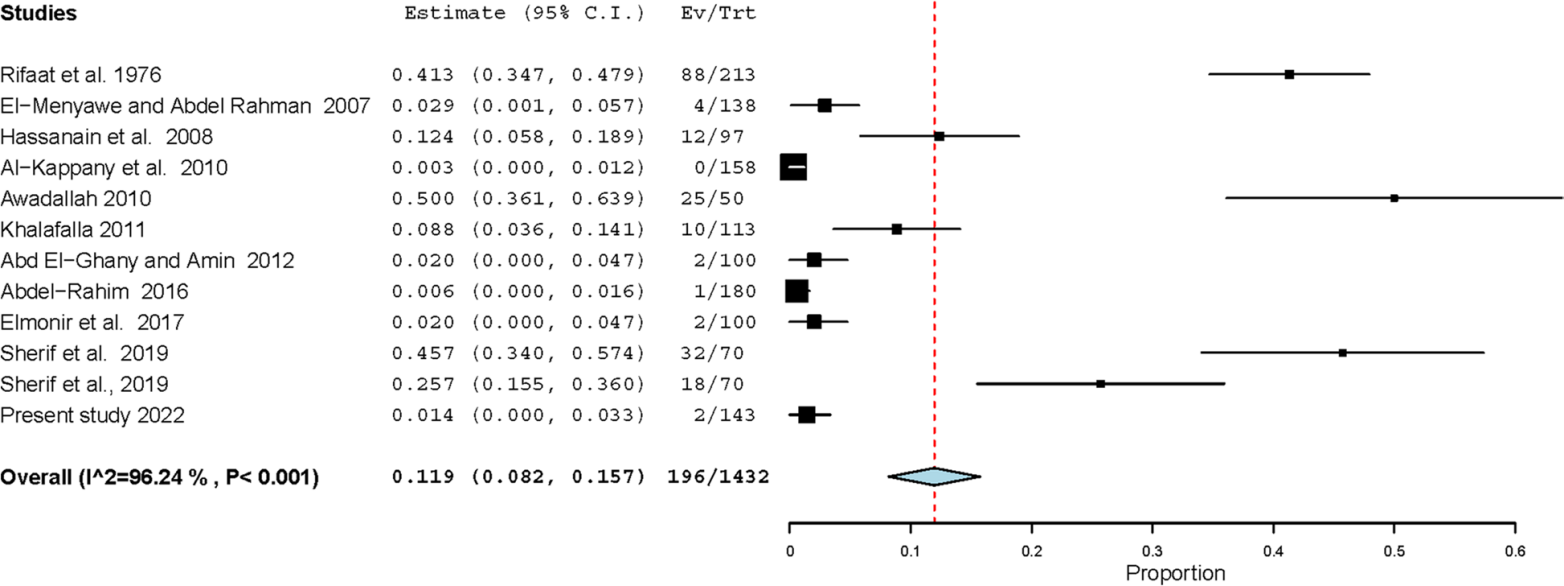
Forest plot diagrams for random effects in the meta-analysis of the prevalence of *T. gondii* infections in cats from Egypt

**Fig. 5:**
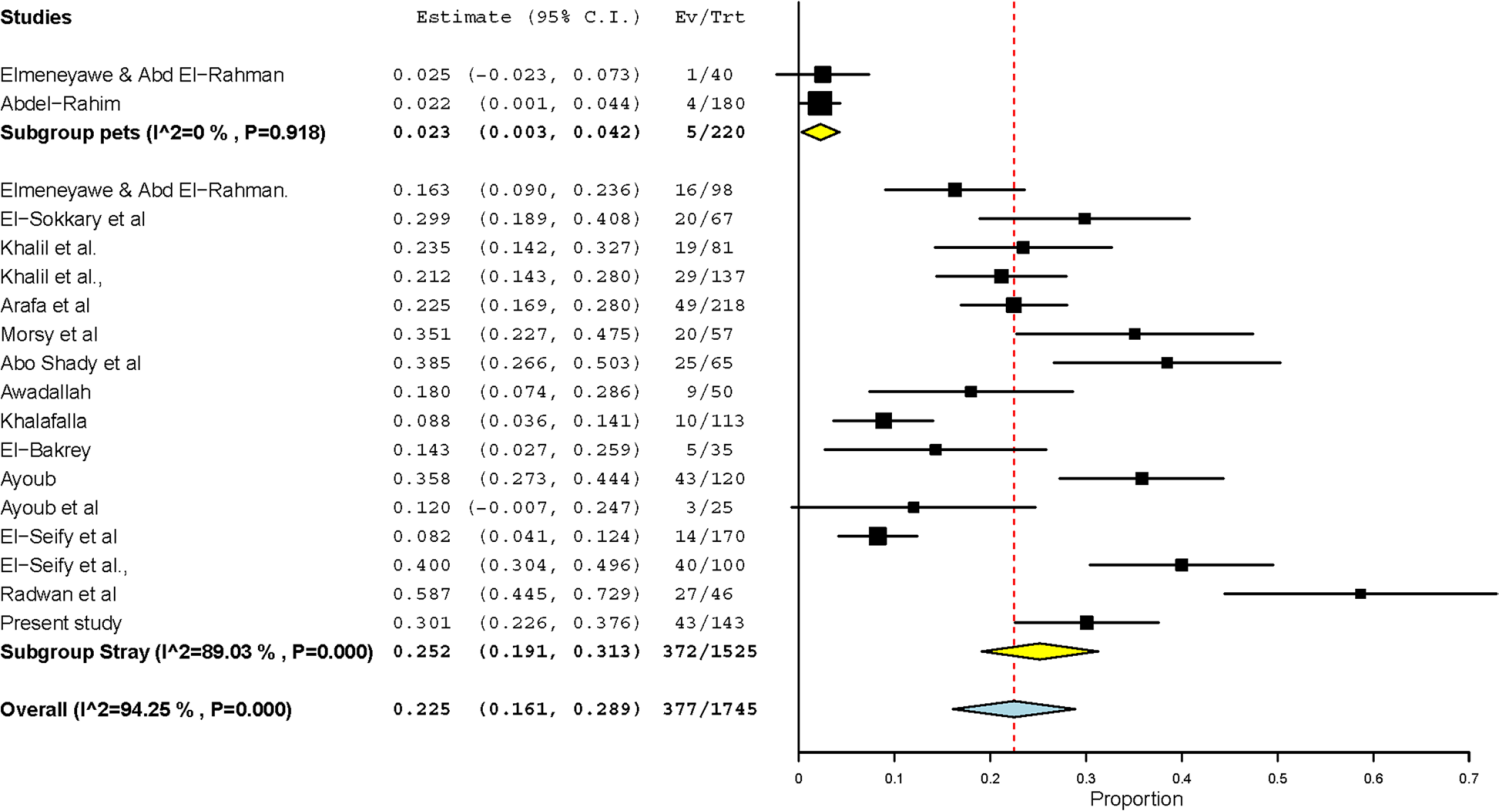
Forest plot diagrams for random effects in the the prevalence of *T. cati* infections in cats from Egypt

**Fig. 6:**
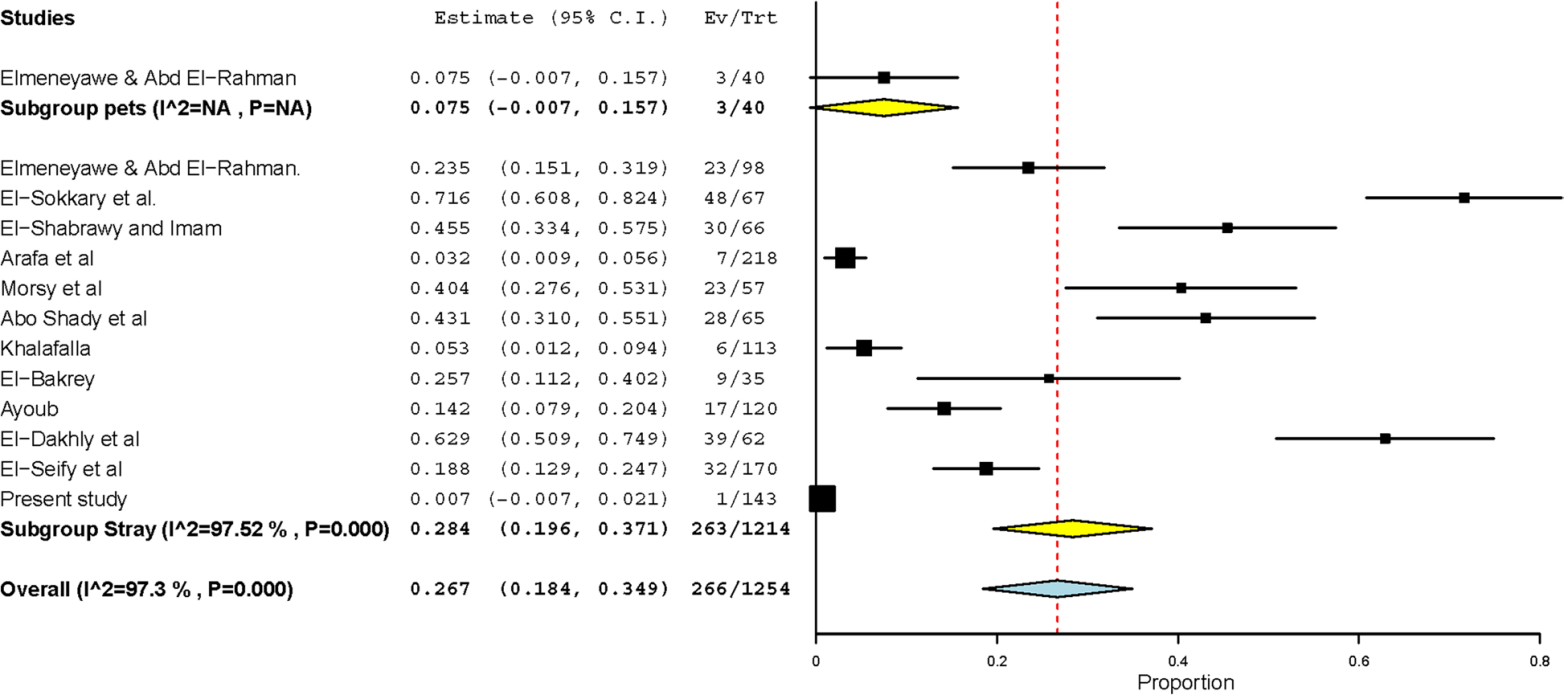
Forest plot diagrams for random effects in the prevalence of *D. caninum* infections in cats from Egypt

Studies describing various GIT protozoa infecting cats in Egypt are scarce with fragmentary results. Findings of these studies on *Toxoplasma gondii* and *Cystoisospora* were included in the meta-analysis conducted considering the very important zoonoses of the former. *Toxoplasma gondii*-like oocysts have been observed in feces of 196 out of 1432 cats tested in 12 datasets, giving rise to a pooled prevalence of 11.9% (95% CI: 8.2 – 15.7%) (Fig. 4). In addition, *T. gondii* antibodies have been detected in sera of 601 out of 1186 cats resulting in a much higher pooled prevalence (49.7%, 25.9 – 73.5%). On the other hand, 21 datasets have described GIT helminths infecting 1866 cats in Egypt (Tables 3, 4). Of these datasets, 11 determined the overall prevalence in 1147 cats, and 683 were found infected with a high pooled prevalence (62.5%, 45.5 – 79.5%). Several helminths were detected after examining either adult worms recovered during intestinal necropsy or eggs detected during faecal examination. Of these helminths, 6 (*T. cati*, *T. leonina,* hookworms, *D. caninum*, taeniids and *Heterophyes heterophyes*) have been frequently observed. Eighteen datasets have observed *T. cati* in 377 out of 1745 cats from various Egyptian governorates, giving rise to a high pooled prevalence (22.5%, 16.1 – 28.9%). The prevalence significantly differed (*p*-value = 0.0346) according to the detection method used; 1277 cats diagnosed via intestinal necropsy had a double prevalence (32.7%) in comparison to 468 cats diagnosed via fecal examination (16.4%). Wild cats (*Felis sylvestris*) have been tested in a single dataset and had also a high *T. cati* prevalence 58.7% (44.5 – 72.9%) (Table 3, Fig. 5). The other Ascarid “*T. leonina”* displayed a lower prevalence (9.5%) in 1128 cats tested in 8 datasets (Table 3). Based on testing of 690 cats in 6 datasets, hookworms had the lowest pooled prevalence (3.2%, 1.2 – 5.1%) among the studied helminths infecting cats in Egypt (Table 3). On the contrary, *D. caninum* displayed the highest pooled prevalence (26.7%, 18.4 – 34.9%); this cestode has been detected in 266 out of 1254 cats tested in 13 datasets (Fig. 6). Like *T. cati*, the prevalence was significantly (*p*-value = 0.0012) higher in 450 cats (44.7%) tested via intestinal necropsy than in 804 cats tested via fecal examination (7.6%) (Table 4). Taeniid eggs and/or adult worms have been observed in 168 out of 1162 cats with a pooled prevalence of 14.4% (8.9 – 20.0%) (Table 4). A similar pooled prevalence (13.1%, 5.8 – 20.3%) was estimated for the trematode parasite “*H. heterophyes*” that has been detected in 69 out of 617 cats tested in 7 datasets (Table 4).

**Table 3:** Pooled prevalence of the most common GIT nematodes detected in cats from Egypt and prevalence variation according to life style, detection method and various Egyptian regions

Parasite/Parameter	No. data sets	No. tested	No. positive	Pooled estimate % based on 95% CI	Heterogeneity *I*^*2*^%
***Toxocara cati***					
Overall prevalence	18	1745	377	22.5 (16.1 – 28.9)	94.25
**Life style**					
Strays	16	1479	345	23.2 (17.6 – 28.9)	87.0
Pets	2	220	5	2.3 (0.3 – 4.2)	0.0
Wild cat (*Felis sylvestris*)	1	46	27	58.7 (44.5 – 72.9)	NA
**Detection method**					
Fecal examination	11	1277	224	16.4 (9.8 – 23.1)	93.75
Necropsy	7	468	153	32.7 (21.9 – 43.6)	85.99
**Region**					
**Middle Egypt**	**9**	**822**	**207**	**24.6 (15.2 – 33.9)**	**92.02**
Cairo	6	477	112	20.3 (8.8 – 31.5)	91.91
Giza	1	81	19	23.5 (14.2 – 32.7)	NA
Cairo, Giza	1	218	49	22.5 (16.9 – 28.0)	NA
Cairo, Fayoum (wild cat)	1	46	27	58.7 (44.5 – 72.9)	NA
**Nile Delta**	**5**	**438**	**107**	**24.6 (12.9 – 36.3)**	**89.15**
Dakahlia	2	208	68	33.0 (25.1 – 40.8)	27.41
Kafr Elsheikh	1	113	10	8.8 (3.6 – 14.1)	NA
Sharkia	1	67	20	29.9 (18.9 – 40.8)	NA
Behera	1	50	9	18.0 (7.4 – 28.6)	NA
**Coastal governorates**	**3** (Alexandria)	**305**	**59**	**20.6 (0.7 – 40.6)**	**94.37**
**Various** (pets)	**1** (Cairo, Giza, Beni-Suef)	**180**	**4**	**2.2 (0.1 – 4.4)**	**NA**
***Toxascaris leonina***					
Overall prevalence	8	1128	107	9.5 (5.3 – 13.7)	88.57
**life style**					
Strays	6	948	100	10.8 (5.6 – 16.0)	89.93
Pets	1	180	7	3.9 (1.1 – 6.7)	NA
**Detection method**					
Fecal examination	6	1009	81	7.3 (3.4 – 11.1)	67.09
Necropsy	2	119	26	20.9 (3.7 – 45.5)	92.04
**Hookworms**					
Overall prevalence	6 (strays)	690	25	3.2 (1.2 – 5.1)	51.62

**Table 4:** Pooled prevalence of the most common GIT flatworms detected in cats from Egypt and prevalence variation according to life style, detection method and various Egyptian regions

Parasite/Parameter	No. data sets	No. tested	No. positive	Pooled estimate % based on 95% CI	Heterogeneity *I*^*2*^%
***Dipylidium caninum***					
Overall prevalence	13	1254	266	26.7 (18.4 – 34.9)	97.30
**Life style**					
Stray	12	1214	263	28.4 (19.6 – 37.1)	97.52
Pet	1	40	3	7.5 (0.7 – 15.7)	NA
**Detection method**					
Fecal examination	6	804	66	7.6 (0.3 – 12.3)	90.6
Necropsy	7	450	200	44.7 (30.5 – 58.9)	90.65
**Region**					
**Middle Egypt**	**6**	**599**	**103**	**21.4 (9.4 – 33.5)**	**94.74**
Cairo	4	315	66	20.5 (9.2 – 31.7)	85.83
Cairo, Giza	2	284	37	23.9 (-17.5 – 65.3)	97.82
**Nile Delta**	**4**	**388**	**83**	**29.2 (9.1 – 49.3)**	**98.57**
Dakahlia	2	208	29	21.4 (-20.1 – 63.0)	97.87
Kafr Elsheikh	1	113	6	5.3 (1.2 – 9.4)	NA
Behera	1	67	48	71.6 (60.8 – 82.4)	NA
**Coastal governorates**	**2** (Alexandria)	**205**	**41**	**19.8 (14.4 – 25.2)**	**0.0**
**Southern Egypt**	**1** (Beni-Suef)	**62**	**39**	**62.9 (50.9 – 74.9)**	**NA**
**Taeniids**					
Overall prevalence	11	1162	168	14.4 (8.9 – 20.0)	93.28
**Life style**					
Stray	10	1122	168	16.1 (9.8 – 22.4)	93.58
Pet	1	0	40	1.2 (-2.1 – 4.6)	NA
**Detection method**					
Fecal examination	6	804	99	11.4 (4.8 – 18.0)	94.62
Necropsy	5	358	69	18.6 (8.6 – 28.6)	86.91
**Region**					
**Middle Egypt**	**5**	**542**	**100**	**18.0 (6.8 – 29.1)**	**94.96**
Cairo	3	258	60	17.7 (2.6 – 38.0)	96.88
Cairo, Giza	2	384	40	19.1 (1.6 – 39.7)	91.98
**Nile Delta**	**4**	**389**	**59**	**16.1 (4.5 – 27.6)**	**92.7**
Dakahlia	2	208	10	4.7 (0.18 – 7.5)	0.0
Kafr Elsheikh	1	114	25	22.1 (14.5 – 29.8)	NA
Behera	1	67	24	35.8 (24.3 – 47.3	NA
**Coastal governorates**	**1** (Alexandria)	**170**	**3**	**1.8 (0.2 – 3.7)**	**NA**
**Sothern Egypt**	**1** (Beni-Suef)	**62**	**6**	**9.7 (2.3 – 17.0)**	**NA**
***Heterophyes heterophyes***					
Overall prevalence	7	617	69	13.1 (5.8 – 20.3)	94.00

## Discussion

The vast majority of cats in Egypt live as strays roaming everywhere and contributing to the occurrence as well as endemicity of several parasites, including those pose potential health risks for humans. The present study is the first to report GIT parasites in stray cats from Gharbia governorate, Egypt, and a high overall prevalence (52.4%) was determined, which is consistent with what have been reported from cats in various Egyptian governorates (see Additional files 2, 3). This prevalence, however, is higher than the 16.4% estimated prevalence for 468 cats tested in 11 earlier Egyptian fecal surveys, which generally used either the sedimentation technique alone or in combination with the flotation technique using the saturated salt solution as the flotation fluid. In the present investigation, samples were tested using both sedimentation and flotation techniques, and a sensitive flotation protocol (modified Wisconsin sucrose flotation) was conducted utilizing centrifugal flotation with the Sheather sugar solution. This is the most effective method to isolate most helminth eggs and coccidial oocysts [24]. In addition, negative samples that had a lot of debris were re-examined.

A wide diverse of parasites was detected and *Opisthorchis*-like eggs were observed for the first time in cats from Egypt. The family Opisthorchiidae includes 33 genera [25], of which *Opisthorchis felineus*, *Opisthorchis viverrini* and *Clonorchis sinensis* are food-borne zoonotic trematodes common to infect fish eating mammals, e.g., dogs, cats and humans. Humans get infected through consumption of raw freshwater fish of the family Cyprinidae, and infected persons often develop fever, diarrhea, recurrent cholangitis, hepatic abscesses and acute pancreatitis [26]. Infections are endemic in Asia as well as in some European countries [27], and no infections have been reported in Africa or the Middle East. However, a case of *C. sinensis* infection has been reported in an Egyptian family, who often consumed imported fish, and diagnosis was based on the characteristic shape of eggs [28]. In Egypt, importing fish is common from various Asian countries to fill the gap in fish production. In the present study, *Opisthorchis*-like eggs were detected in only one out of 143 cats tested, probably that cat got infected through consumption of imported fish. To suggest Egypt as a new geographical range for the occurrence of *Opisthorchis* infections, a large-scale investigation on cats from various governorates is required, and should include PCR testing for *Opisthorchis*-positive samples if present. Regarding the other GIT parasites detected in the present study, results will be discussed in the following sections in the context of findings of the meta-analysis conducted to evaluate the parasitic zoonoses from cats in Egypt.

### GIT helminths detected in cats from Egypt

Analysis of findings of 11 datasets that have determined the overall prevalence of GIT helminths in 1147 cats surveyed in Egypt, displayed a very high pooled prevalence (62.5%, 45.5 – 79.5%). Given that most of the sampled cats lived as strays, findings of this analysis confirm the widespread helminthic infections in stray cats likely due inadequate control measures as well as easy access to the intermediate hosts [3]. A wide diverse of GIT helminths have been found infecting cats from Egypt; of them, data on 6 were found suitable for conducting the meta-analysis.

## Round worms

*Toxocara cati* is one of the most prevalent GIT parasites in cats worldwide [12]. Consistent with earlier surveys from Egypt, the parasite was the most frequently detected (30.0%) in 143 fecal samples from cats in Gharbia. Infections have been observed in cats from various Egyptian governorates; nonetheless, no *T. cati* has been found in 62 necropsied cats from Beni-Suef governorate [29]. Number of cats recruited varied among studies with the majority being from Cairo and Giza, which may represent a limitation for the meta-analysis conducted in the present study. Overall, the estimated pooled prevalence for *T. cati* infections in 1745 cats tested in 18 datasets from Egypt was higher (22.5%) than that reported globally (17.0%; [12]). The later included only 2 datasets from Egypt that have tested 283 cats with a prevalence of 8.5%. In Egypt, most cats are strays and can disseminate *T. cati* eggs everywhere in the environment; *Toxocara* eggs have been frequently recovered from soil in Egypt with a high burden up to 13–19 eggs/10 g of soil [30]. Therefore, the high *Toxocara* prevalence in the Egyptian cats suggests the underestimated role of *T. cati* in human toxocarosis in Egypt, where the disease is quite common [13].

The genus *Toxascaris* comprises a single species “*T. leonina*” that can infect both dogs and cats with limited pathologies in comparison to members of the genus *Toxocara*. In contrary to earlier surveys from Egypt, an unexplained high prevalence (22.4%) of *T. leonina* eggs was detected in 143 fecal samples tested from cats in Gharbia. Overall, the pooled prevalence estimated for this parasite in 1128 cats from Egypt (9.5%) was approximately 3 times higher than that estimated globally (3.4%), but coincided with that has been estimated for cats in the Eastern Mediterranean region (10.0%) where Egypt is located [31]. Because *T. leonina* has a limited zoonotic potential, its high prevalence in cats has a little significance for humans.

Likewise, the 143 fecal samples tested from cats in Gharbia has a higher prevalence of hookworm eggs (8.4%) than that have been reported from cats in the other Egyptian governorates (see Additional file 2). Hookworms are common to infect humans worldwide with various routes of transmission, which emphasizes the significance of our findings. The estimated pooled prevalence of hookworm infections in cats from Egypt was 3.2%. Variable prevalences have been reported from cats worldwide, and a recent study have determined a high pooled prevalence (26.0%) in cats from Asia [32]. Cats can be infected with various hookworms including *Ancylostoma tubaeforme*, *Ancylostoma braziliense*, *Ancylostoma ceylanicum* and *Uncinaria stenocephala*, with the former is the globally predominant species in cats [33]. A few *Ancylostoma caninum* (the most predominant dog species) cases have been reported from cats worldwide [34]. However, *A. caninum* was proposed as the species present in most studies on cats from Egypt (see Additional file 2).

## Cestodes

*Dipylidium caninum* is a ubiquitous cestode that can infect dogs and cats. In the present study, a single sample (0.7%) out of the 143 tested in Gharbia had *D. caninum* egg capsules, which can be explained by infrequent discharge of egg capsules outside *D. caninum* gravid proglottids resulting in underestimated infections determined via the routine microscopic examination of feces [19]. This was also evidenced when the subgroup analysis was conducted to detect the variation in *D. caninum* prevalence in cats from Egypt according to the detection method used. Cats that were tested via the intestinal necropsy to detect the adult worms had significantly higher infections (*p*-value = 0.0012) than those tested via the fecal examination (Table 3). In total, high *D. caninum* pooled prevalence (26.7%, 18.4 – 34.9%) was detected in 1254 cats tested in 13 datasets from various Egyptian governorates, which identify cats as a major risk for *D. caninum* infections in humans from Egypt. However, the parasite displayed low prevalence in children in Egypt [35]. Likewise, a few human cases, mostly in children, have been reported worldwide [36].

Contrastingly to *D. caninum*, *Taenia* spp. eggs are commonly expelled outside the gravid proglottids during animal defecation [19]. This strengthens the validity of fecal examination to detect taeniid eggs, which were observed in 4.2% (6) of the 143 samples tested in Gharbia. The overall pooled prevalence of *Taenia* spp. detected in 1162 cats from various Egyptian governorates was 14.4% (8.9 – 20.0%). *Taenia taeniaeformis* is the only known species that can infect cats, and the parasite has been frequently detected in earlier surveys from Egypt (see Additional file 2). *Taenia taeniaeformis* larvae (strobilocercus fasciolaris) have been detected in 11 out of 120 rats from Egypt [37]. On the other hand, a report by El-Bakrey [38] is interesting. The author examined intestinal contents of 35 stray cats in Alexandria governorate, and *Echinococcus* spp. have been detected in 3 cats. It is well known that cats cannot serve as definitive hosts for *E. granulosus*, but *Echinococcus multilocularis*, the etiological agent of alveolar echinococcosis in humans. *Echinococcus multilocularis* is widespread in the northern hemisphere, and no *E. multilocularis* infections have been reported from Egypt; however, the parasite has been reported in other neighbouring countries in North Africa including Tunisia and Morocco [39].

## Trematodes

Heterophyids are common global zoonoses from dogs and cats. No Heterophyid eggs were noticed in any of the 143 cat fecal samples tested in Gharbia; however, *Heterophyes heterophyes* has been observed in cats from various Egyptian governorates and displayed a considerably high pooled prevalence (13.1%, 5.8 – 20.3%). A high prevalence of heterophyid metacercariae (32.0%) has been detected in 100 Tilapia fishes from Northern Egypt, and heterophyid eggs have been observed in stools of 10 (13.3%) out of 75 residents from this region [40].

### GIT protozoa detected in cats from Egypt

Various GIT protozoa have been detected in cats from Egypt including *Toxoplasma gondii*, the most important zoonotic protozoan transmissible from cats. The parasite can cause serious disease in humans particularly immunocompromised patients and unborn infants [41], and *T. gondii* infections appear highly prevalent in animals and humans from Egypt [42]. Infected cats shed millions of *T. gondii* oocysts in feces for relatively short time (1–3 weeks) during their life, then become immune and seldom to shed *T. gondii* oocysts again [41]. This explains the scarce detection of *T. gondii* oocysts in feces of cats. In a recent survey on 24,000 cats from Europe, *T. gondii*-like oocysts have been observed in only 0.2% [43]. However, some earlier reports on cats from Egypt stated very high prevalences up to 50.0% (see Additional file 3). Results of the meta-analysis conducted in the present study for *T. gondii* oocysts in cats from Egypt revealed a 5 times higher pooled prevalence (11.9%) than that estimated for cats worldwide (2.6%, [44]). Nonetheless, the estimated pooled prevalence for *T. gondii* antibodies in sera of cats from Egypt (49.7%) is not far from that estimated for cats globally (37.5%, [44]), which suggests the overestimation of *T. gondii* oocysts in cats from Egypt. To resolve this debate, feces of 143 stray cats from Gharbia, Egypt were carefully examined in the present study, and a few (3–5) *T. gondii*-like oocysts were observed in only 2 (1.4%) samples. Oocysts of *Toxoplasma*, *Hammondia* and *Besnoitia* in cat feces are quite similar morphologically, and the differential diagnosis requires further procedures particularly mouse inoculation assays [41], that were not conducted in the present study. It is worthy mentioned that many oocysts as smaller as 12 µm were observed in 10 out of the 143 samples tested. These oocysts had micropyles and belonged to the genus *Eimeria*, thus were identified as spurious parasites. In many rural areas in Egypt, household keeping of birds is common, and residents usually slaughter and eviscerate those birds in-home, and the viscera are thrown in the streets to feed stary cats and dogs. The existence of *Eimeria* oocysts in feces of cats is a possible cause for the overestimation of *T. gondii* oocysts. Noteworthy, *Toxoplasma gondii* oocysts were not detected in rectal contents of 158 cats that were tested in Dr Dubey Lab at USDA to detect *T. gondii* genotypes in various tissues of stray cats from Giza, Egypt [45].

Many reports documented *Cystoisospora* (formerly *Isospora*) infections in a total of 1220 cats from Egypt, with an estimated pooled prevalence of 14.1% (8.3 – 19.8%), which is much lower than that has been determined in the only report published from Africa on 103 cats in Kenya (43.7%, [7]). Variable prevalences (occasionally up to 84.0%) have been documented in cats worldwide [46]. Cats can be infected with 2 *Cystoisospora* spp., *C. felis* and *C. rivolta*, and the latter is more pathogenic and can cause diarrhea associated with villar atrophy and cryptitis in newborn kittens [46]. In Egypt, an outbreak of diarrhea in 3–5 weeks old kittens infected with *Cystoisospora* spp. has been documented [47]. However, the present study is the first to report *C. rivolta* in cats from Egypt; the parasite was detected in 4.9% out of 143 fecal samples from cats in Gharbia governorate. On the other hand, cats serve as definitive hosts for many *Sarcocystis* spp. that utilize various herbivores as intermediate hosts [48]. Of the 143 cats tested in Gharbia, sporocysts of *Sarcocystis* spp. were detected in 10 (7.0%). This prevalence is high when compared to that determined in 2 earlier reports from Egypt (see Additional file 3). However, sarcocystosis is common among ruminants in Egypt and *Sarcocystis fusiformis*, which circulates in a cat-buffalo cycle, is prevalent among water buffaloes in Egypt causing significant economic losses [49]. Scarce reports on some other protozoal infections (e.g., *Cryptosporidium* and *Giardia*) are also available from cats in Egypt. It seems that cats have a minimized role in the zoonotic cycles of both protozoa [50, 51].

## Conclusion

High prevalence rates of zoonotic GIT parasites of cats in Egypt are alarming since the majority of cats live as strays in this country, which highlights the urgent need for implementing effective control strategies. Toxocariosis is likely the most important parasitic zoonosis from cats in Egypt due to their high prevalence and frequent detections. *Toxoplasma gondii* prevalence in cats is questionable and the high prevalence may be attributed to misdiagnosis with other protozoan oocysts of relatively small sizes. *Opisthorchis*-like eggs detected in this study may indicate zoonotic hazards of imported raw fish. A possible bias in results of the meta-analysis conducted in the present study may come from the high heterogeneity between the included studies, which have not also covered some governorates particularly the southern ones.

## Supplementary Information

**Additional file 1:** **Table S1.** Patterns of the mixed GIT parasitic infections detected in 143 faecal samples of stray cats from Gharbia governorate, Egypt.

**Additional file 2:** **Table S2.** Reports on prevalence of GIT helminths in cats from various governorates in Egypt.

**Additional file 3:** **Table S3.** Reports on GIT protozoa detected in feces of cats from various governorates in Egypt.

## Data Availability

The datasets supporting the conclusions of this article are included within the article (and its additional files).
