# Supplementary material for: Gastrointestinal parasites of cats in Egypt: high prevalence high zoonotic risk
Source: BMC Vet Res. 2022 Nov 29;18:420. doi: 10.1186/s12917-022-03520-0 (PMC9706847; doi:10.1186/s12917-022-03520-0)
Supplement: Supplementary file 1 — Additional file 1: Table S1. Patterns of the mixed GIT parasitic infections detected in 143 faecal samples of stray cats from Gharbia governorate, Egypt. [file 12917_2022_3520_MOESM1_ESM.docx]

**Table S1**. Patterns of the mixed GIT parasitic infections detected in 143 faecal samples of stray cats from Gharbia governorate, Egypt.

| Infection forms | Frequency | % Prevalence |
| --- | --- | --- |
| **Double**  T. cati +T. leonina | 12 | 8.4 |
| T. cati + hookworms | 1 | 0.7 |
| T. cati + taeniids | 1 | 0.7 |
| T. cati + C. rivolta | 1 | 0.7 |
| T. cati + Sarcocystis | 2 | 1.4 |
| T. leonina + hookworms | 1 | 0.7 |
| T. leonina + taeniids | 1 | 0.7 |
| T. leonina + C. felis | 1 | 0.7 |
| Hookworms + Strongyloides spp. | 1 | 0.7 |
| Hookworms+ C. rivolta | 1 | 0.7 |
| Taeniids + C. felis | 1 | 0.7 |
| Taeniids + Sarcocystis | 1 | 0.7 |
| Physaloptera spp. + Sarcocystis | 1 | 0.7 |
| C. felis + Sarcocystis | 1 | 0.7 |
| C. rivolta + Sarcocystis | 1 | 0.7 |
| Alaria spp. + Opisthorchis-like eggs | 1 | 0.7 |
| **Triple**  T. cati + D. caninum + Alaria spp. | 1 | 0.7 |
| T. cati + T. leonina + hookworms | 1 | 0.7 |
| T. cati + T. leonina + C. rivolta | 1 | 0.7 |
| T. cati + T. leonina + Sarcocystis | 1 | 0.7 |
| T. cati + hookworms + Sarcocystis | 1 | 0.7 |
| T. leonina + hookworms + C. rivolta | 1 | 0.7 |
| T. leonina + hookworms + T. gondii-like oocyst | 1 | 0.7 |
| T. leonina + C. felis + C. rivolta | 1 | 0.7 |
| **Quadruple**  T. cati + T. leonina + C. felis + T. gondii-like oocyst | 1 | 0.7 |
