# Supplementary material for: Gastrointestinal parasites of cats in Egypt: high prevalence high zoonotic risk
Source: BMC Vet Res. 2022 Nov 29;18:420. doi: 10.1186/s12917-022-03520-0 (PMC9706847; doi:10.1186/s12917-022-03520-0)
Supplement: Supplementary file 2 — Additional file 2: Table S2. Reports on prevalence of GIT helminths in cats from various governorates in Egypt. [file 12917_2022_3520_MOESM2_ESM.docx]

**Table S2**. Reports on prevalence of GIT helminths in cats from various governorates in Egypt

| **Governorate** | **No. tested (life style)** | **Identified stage, method** | **Overall prevalence%** | **Commonly identified helminths (%)** | **Other detected helminths** | **Reference** |
| --- | --- | --- | --- | --- | --- | --- |
| Giza | 81, stray | Eggs, FE | -- | *T. cati* (23.0) |  | Khalil et al. (1976) |
| Cairo | 137, stray |  |  | *T. cati* (21.0) |  |  |
| Cairo, Giza | 218, stray | Eggs, FE | 36.7 | *T. cati (*22.5), *A. caninum* (1.4), *T. taeniaeformis* (9.2), *D. caninum* (3.2), *H. heterophyes* (1.4) | *Diplopylidium* spp. (2.7), *Joyeuxiella* spp. (0.9), *Asocotyle* spp. (1.4), *Euparadistomum* (3.2), *Gnathostomata* spp. (0.5) | Arafa et al. (1978) |
| Cairo, Giza | 66, stray | Adult, necropsy | 77.3 | *T. taeniaeformis* (30.3), *D. caninum* (45.4) | *Diplopylidium acanthotretum, Diplopylidium nolleri, Joyeuxiella* | El-Shabrawy and Imam (1978) |
| Cairo | 57, stray | Adult, Necropsy | 71.9 | *T. cati* (35.1), *T. leonina* (8.8), *A. caninum* (5.3), *D. caninum* (40.3), *H. heterophyes* (7.0) | *Diplopylidium* spp., *Trichinella spiralis* | Morsy et al. (1981) |
| Dakahlia | 65, stray | Adult, Necropsy | -- | *T. cati* (38.5), *T. taeniaeformis* (6.2), *D. caninum* (43.1), *H. heterophyes* (21.5) | *Haplorchis yokogawai*, *Pygidiopsis* *genata*, *Prohemistomum* *vivax*, *Echinocasmus* *perfoliatus* | Abo Shady et al. (1983) |
| Behera | 67, stray | Adult, Necropsy | 100 | *T. cati* (29.8), *A. caninum* (4.5), *T. taeniaeformis* (35.8), *D. caninum* (71.7), *H. heterophyes* (56.7) | *T. spiralis*, *P.* *vivax*, *Phagicola longicollis* | El Sokkary et al. (1987) |
| Cairo | 40, stray | Eggs, FE | 22.5 | *T. cati* (2.5)*, D. caninum* (7.5) |  | El Menyawe and Abdel Rahman (2007) |
|  | 98, stray | Adult, necropsy | 47.9 | *T. cati* (16.3), *Taenia spp.* (15.3), *D. caninum* (23.5) |  |  |
| Sharkia | *50, stray | Eggs, FE | 60.0 | *T. cati* (18.0), *Strongyloides* spp. (2.0) |  | Awadallah (2010) |
| Kafr Elsheikh | *113, stray | Eggs, FE | 91.0 | *T. cati* (9.0), *T. leonina* (5.0), *A. tubaeforme* (4.0), *T. taeniaeformis* (22.0), *D. caninum* (5.0), *H. heterophyes* (3.0), | *Capillaria* spp., *Linguatula serrata* | Khalafalla (2011) |
| Alexandria | 35, stray | Adult, necropsy | NS | *T. cati* (14.2)*, A. caninum* (2.8)*, H. heterophyes* (11.4), *D. caninum* (25.7) | *Echinococcus spp*. (8.6) | El-Bakrey (2012) |
| Qena | 39, stray | Adult, necropsy | -- | *P. praeputialis* (71.2) |  | Mohamadain and Ammar (2012) |
| Cairo | 120, stray | Eggs/oocysts, FE | 57.5 | *T. cati* (35.8), *T. leonina* (1.7), *T. taeniaeformis* (37.5), *D. caninum* (14.2) | *C. hepatica* | Ayoub (2014) |
| NS | 25, stray | Eggs, FE | -- | *T. cati* (12.0) |  | Ayoub et al. (2015) |
| Cairo, Giza, Beni-Suef | 180, household | Eggs/oocysts, FE |  | *T. leonina* (3.9), *Ascaris* spp. (4.4) |  | Abdel-Rahim (2016) |
| Beni-Suef | 62, stray | Adult, Necropsy | 87.0 | *T. leonina* (33.8), *T. taeniaeformis* (9.6), *D. caninum* (62.9), *H. heterophyes* (3.2), | *Pygidiopsis summa*, *Heterophyes nocens*, *Echinochasmus liliputanus*, *Alaria* spp., *P. varium*, *Ascocotyle* spp., *Haplorchis* spp., *P. vivax*, *Euparadistomum herpestesi*, *D. acanthoterta*, *D. nolleri*, *Joyeuxiella* spp., *Anisakes simplex* | El-Dakhly et al. (2017) |
| Alexandria | *170, stray | Eggs, FE | 58.2 | *T. cati* (8.2), *T. leonina* (8.3), *Physaloptera* spp. (0.6), *S. cati* (0.6), hook worms (1.8), *T. taeniaeformis* (1.8), *D. caninum* (18.8) | *Hymenolepis* spp., *Trichuris* spp., Heterophyids, *Alaria* spp., *Mesostepahnus* spp. | El-Seify et al. (2017) |
| Alexandria | 100, stray | Adult, necropsy | -- | *T. cati* (40.0) |  | El-Seify et al. (2021) |
| Gharbia | 143, stray | Eggs/oocysts, FE | 52.4 | *T. cati* (30.0), *T. leonina* (22.4), Hookworms (8.4), *Strongyloides* spp. (2.1), *Physaloptera* spp. (2.1), Taeniid eggs (4.2), *D. caninum* (0.7) | *Alaria* spp. (1.4), Opisthorchis-like (0.7) | Present study |

**FE,** fecal examination

*fecal samples from cats and collected from the environment

**References**

Abdel-Rahim, M.M. 2016. Public health importance of enteric parasites of pet dogs and cats. Thesis (M.S.), Beni-Suef University, Egypt, pp 110.

Abo-Shady, A.F., Ali, M.M. and Abdel-Magied, S., 1983. Helminth parasites of cats in Dakahlia, Egypt. Journal of the Egyptian Society of Parasitology, 13(1), pp.129-133.

Arafa, M.S., Nasr, N.T., Khalifa, R., Mahdi, A.H., Mahmoud, W.S. and Khalil, M.S., 1978. Cats as reservoir hosts of Toxocara and other parasites potentially transmissible to man in Egypt. Acta Parasitologica Polonica, 25, pp.383-389.

Awadallah, M.A., 2010. Endoparasites of zoonotic importance. Global Veterinaria, 5(6), pp.348-355.

Ayoub, M.B., 2014. Parasitic infection in stray cats and dogs with special reference to ultrastructure of the recovered worms. Animal Health Research Journal, 2 (3), pp. 165-178

Ayoub, M.B., Hamed, H.R., and Ab El -Soad, S.N. 2015. Value of ELISA in diagnosis of toxocariasis in pets animals using larval antigens. Animal Health Research Journal, 3 (1), pp. 263-271.

El Menyawe, S.M. and Abdel Rahman M.A.M. 2007. The role of dogs and cats in transmitting some parasites to man in Cairo governorate. Egyptian Veterinary Medical Society of Parasitology Journal (EVMSPJ), IV (1), pp.735-755.

El Sokkary, M.Y., Heikal, F.A., Abdel Rahman, M.S., 1987. Helminths of stray cats in Behera Governorate with special reference to their zoonotic importance. Alexandria Journal of Veterinary Sciences [AJVS]; 3 (2): 119-131.

El-Bakrey, K.M., 2012. Investigation on some internal parasites affecting stray dogs and cats. Alexandria Journal of Veterinary Sciences [AJVS]; 35 (1), 211–219.

El-Dakhly, K.M., Aboshinaf, A.M., El-Nahass, E.S. and Gharib, A.E.T.F., 2017. A preliminary study on the helminth fauna in necropsied stray cats (Felis catus) in Beni-Suef, Egypt. Journal of Advanced Veterinary Research, 7(4), pp.87-92.

El-Shabrawy, M.N. and Imam, E.A., 1978. Studies on cestodes of domestic cats in Egypt with particular reference to species belonging to genera Diplopylidium and Joyeuxiella. J Egypt Vet Med Assoc, 38(4), pp.19-27.

El-Seify, M.A., Aggour, M.G., Sultan, K. and Marey, N.M., 2017. Gastrointestinal helminths of stray cats in Alexandria, Egypt: A fecal examination survey study. Veterinary Parasitology: Regional Studies and Reports, 8, pp.104-106.

El-Seify, M.A., Marey, N.M., Satour, N., Elhawary, N.M. and Sultan, K., 2021. Prevalence and Molecular Characterization of Toxocara cati Infection in Feral Cats in Alexandria City, Northern Egypt. Iranian Journal of Parasitology, 16(2), p.270.

Khalafalla, R.E., 2011. A survey study on gastrointestinal parasites of stray cats in northern region of Nile delta, Egypt. PLoS One, 6(7), p.e20283.

Khalil HM, Khaled ML, Arafa MS, Sadek MS. Incidence of Toxocara canis and Toxocara cati infections among stray dogs and cats in Cairo and Giza Governorates, A.R.E. J Egypt Public Health Assoc. 1976;51(1):45-9.

Mohamadain, H.S. and Ammar, K.N., 2012. Redescription of Physaloptera praeputialis von Linstow, 1889 (Nematoda: Spirurida) infecting stray cats (Felis catus Linnaeus, 1758) in Qena, Egypt and overview of the genus taxonomy. J Egypt Soc Parasitol, 42, pp.675-690.

Morsy, T.A., Sadek, M.S. and Hamid, M.A., 1981. Intestinal parasites of stray cats in Cairo, Egypt. Journal of the Egyptian Society of Parasitology, 11(2), pp.331-345.
