# Supplementary material for: Gastrointestinal parasites of cats in Egypt: high prevalence high zoonotic risk
Source: BMC Vet Res. 2022 Nov 29;18:420. doi: 10.1186/s12917-022-03520-0 (PMC9706847; doi:10.1186/s12917-022-03520-0)
Supplement: Supplementary file 3 — Additional file 3: Table S3. Reports on GIT protozoa detected in feces of cats from various governorates in Egypt. [file 12917_2022_3520_MOESM3_ESM.docx]

**Table S3**. Reports on GIT protozoa detected in feces of cats from various governorates in Egypt.

| **Region** | **No. examined /mode of life** | **Identified protozoa (%)** | **Reference** |
| --- | --- | --- | --- |
| Giza | 81, stray | *Isospora* spp. (29.9) | Khalil et al. (1976) |
| Cairo | 137, stray | *Isospora* spp. (8.0) |  |
| Cairo, Giza | 213, stray | *Isospora* spp. (12.2), ***T. gondii*** (41.3) | Rifaat et al. (1976) |
| Cairo, Giza | 218, stray | *I. felis* (16.1) | Arafa et al. (1978) |
| Cairo | 57, stray | *I. felis* (7.0) | Morsy et al. (1981) |
| Qalubiya |  | *Giardia* spp. (11.0) | Khalifa (1999) |
| Cairo | 138, stray | *I. felis* (16.7), ***T. gondii*** (2.9), *Sarcocystis* (1.4), *Entamoeba histolytica* (26.8) | El Menyawe and Abdel Rahman (2007) |
| Cairo, Giza, Qalubiya | 97, stray, housed | ***T. gondii*** (12.3) | Hassanain et al. (2008) |
| Giza | 158, stray | ***T. gondii*** (0.0) | Al-Kappany et al. (2010) |
| Sharkia | 50, stray | ***T. gondii*** (50.0), *Cryptosporidium* (30.0) | Awadallah (2010) |
| Kafr Elsheikh | 113, stray | *Isospora* spp. (1.8), ***T. gondii*** (8.8), *Sarcocystis* (0.9), *Giardia* (1.8) | Khalafalla (2011) |
| Sharkia | 100, stray | ***T. gondii*** (2.0)* | Abd El-Ghany and Amin (2012) |
| Alexandria | 35, stray | *Cryptosporidium* (22.8) | El-Bakrey (2012) |
| Cairo | 120, stray | *Isospora* spp. (31.7) | Ayoub (2014) |
| Cairo, Giza, Beni-Suef | 180, household | ***T. gondii*** (0.5), *Cryptosporidium* (6.7), *E. histolytica* (5.5) | Abdel-Rahim (2016) |
| Kafr Elsheikh | 100, stray | ***T. gondii*** (2.0) | Elmonir et al. (2017) |
| Different | 70, stray | ***T. gondii*** (45.7)^1^ | Sherif et al. (2019) |
|  | 70, household | ***T. gondii*** (25.7)^2^ |  |
| Gharbia | 143, stray | *I. felis* (4.2)*, I. rivolta* (4.9)*,* ***T. gondii-like* (1.4)*,*** *Sarcocystis* spp. (7.0) | Present study |

^1^Of them, 18 samples were PCR- positive for *T. gondii*.

^2^Of them, 8 samples were PCR- positive for *T. gondii*.

*The recovered *T. gondii* oocysts two from naturally infected cats were molecularly confirmed in B1 gene-PCR reactions.

**References**

Abd El-Ghany, A.M. and Amin, M.A.M. 2012. Epidemiology and molecular detection of zoonotic Toxoplasma gondii in cat feces and seroprevalence of anti-Toxoplasma gondii antibodies in pregnant women and sheep. Life Science Journal, 9(1s).

Abdel-Rahim, M.M. 2016. Public health importance of enteric parasites of pet dogs and cats. Thesis (M.S.), Beni-Suef University, Egypt, pp 110.

Al-Kappany, Y.M., Rajendran, C., Ferreira, L.R., Kwok, O.C.H., Abu-Elwafa, S.A., Hilali, M. and Dubey, J.P., 2010. High prevalence of toxoplasmosis in cats from Egypt: isolation of viable Toxoplasma gondii, tissue distribution, and isolate designation. Journal of Parasitology, 96(6), pp.1115-1118.

Arafa, M.S., Nasr, N.T., Khalifa, R., Mahdi, A.H., Mahmoud, W.S. and Khalil, M.S., 1978. Cats as reservoir hosts of Toxocara and other parasites potentially transmissible to man in Egypt. Acta Parasitologica Polonica, 25, pp.383-389.

Awadallah, M.A., 2010. Endoparasites of zoonotic importance. Global Veterinaria, 5(6), pp.348-355.

Ayoub, M.B., 2014. Parasitic infection in stray cats and dogs with special reference to ultrastructure of the recovered worms. Animal Health Research Journal, 2 (3), pp. 165-178

El Menyawe, S.M. and Abdel Rahman M.A.M. 2007. The role of dogs and cats in transmitting some parasites to man in Cairo governorate. Egyptian Veterinary Medical Society of Parasitology Journal (EVMSPJ), IV (1), pp.735-755.

El-Bakrey, K.M., 2012. Investigation on some internal parasites affecting stray dogs and cats. Alexandria Journal of Veterinary Sciences [AJVS]; 35 (1), 211–219.

Elmonir, W., Harfoush, M.A., El-Tras, W.F. and Kotb, S.A., 2017. Toxoplasmosis in stray cats and pregnant women in Egypt: Association between socio-demographic variables and high-risk practices by pregnant women. Life Science Journal, 14(10).

Hassanain, M.A., Barakat, A.M., ELfadaly, H.A., Hassanain, N.A. and Shaapan, R.M., 2008. Zoonotic impact of Toxoplasma gondii sero-prevalence in naturally infected Egyptian kittens. Journal of the Arab Society for Medical Research, 3(2), pp.243-248.

Khalafalla, R.E., 2011. A survey study on gastrointestinal parasites of stray cats in northern region of Nile delta, Egypt. PLoS One, 6(7), p.e20283.

Khalil HM, Khaled ML, Arafa MS, Sadek MS. Incidence of Toxocara canis and Toxocara cati infections among stray dogs and cats in Cairo and Giza Governorates, A.R.E. J Egypt Public Health Assoc. 1976;51(1):45-9.

Khalifa NO. Prevalence of Giardia cysts in pets, rodents, with possible implications in infantile diarrhea in Kaliobia Governorate. Vet Med J Giz 1999; 47:59–65.

Morsy, T.A., Sadek, M.S. and Hamid, M.A., 1981. Intestinal parasites of stray cats in Cairo, Egypt. Journal of the Egyptian Society of Parasitology, 11(2), pp.331-345.

Rifaat MA, Arafa MS, Sadek MS, Nasr NT, Azab ME, Mahmoud W, Khalil MS. Toxoplasma infection of stray cats in Egypt. J Trop Med Hyg. 1976 Mar;79(3):67-70.

Sherif, H.R., Gerges, A.A., Elsify, A. and Hadad, G.A., 2019. Molecular and Serological Survey for Toxoplasmosis in Cats from an urban populations in Egypt. Alexandria Journal for Veterinary Sciences, 63(1), pp. 26-30.
